# Multi-Calib: A Scalable LiDAR–Camera Calibration Network for Variable Sensor Configurations

**DOI:** 10.3390/s25237321

**Published:** 2025-12-02

**Authors:** Leyun Hu, Chao Wei, Meijing Wang, Zengbin Wu, Yang Xu

**Affiliations:** 1 School of Mechanical Engineering, Beijing Institute of Technology, Beijing 100081, China; 2 China North Vehicle Research Institute, Beijing 100072, China

**Keywords:** multi-LiDAR–camera calibration, deep learning, cross-modal channel-wise attention

## Abstract

Traditional calibration methods rely on precise targets and frequent manual intervention, making them time-consuming and unsuitable for large-scale deployment. Existing learning-based approaches, while automating the process, are typically limited to single LiDAR–camera pairs, resulting in poor scalability and high computational overhead. To address these limitations, we propose a lightweight calibration network with flexibility in the number of sensor pairs, making it capable of jointly calibrating multiple cameras and LiDARs in a single forward pass. Our method employs a frozen pre-trained Swin Transformer as a shared backbone to extract unified features from both RGB images and corresponding depth maps. Additionally, we introduce a cross-modal channel-wise attention module to enhance key feature alignment and suppress irrelevant noise. Moreover, to handle variations in viewpoint, we design a modular calibration head that independently estimates the extrinsics for each LiDAR–camera pair. Through large-scale experiments on the nuScenes dataset, we show that our model, requiring merely 78.79 M parameters, attains a mean translation error of 2.651 cm and a rotation error of $0.246^{\circ }$, achieving comparable performance to existing methods while significantly reducing the computational cost.

## 1. Introduction

LiDAR and cameras constitute the primary sensing modalities in intelligent perception, as their capabilities complement one another. LiDAR point clouds provide accurate spatial structure and precise 3D position, while camera images offer dense appearance information and rich semantic contexts. Effectively combining these two modalities significantly boosts performance for downstream applications, including object detection [1,2,3] and semantic segmentation [4,5,6]. However, the benefits of multi-modal fusion hinge critically on the quality of extrinsic calibration. A precise calibration matrix is essential for accurate spatial alignment between the 3D point clouds and the 2D images, enabling reliable fusion at both data and feature levels.

**Figure 1:**
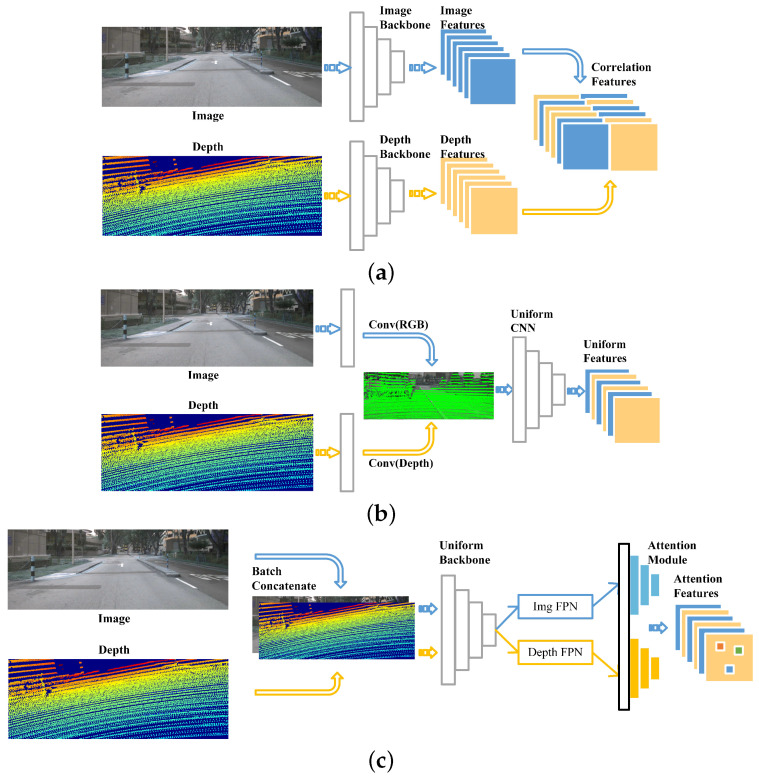
Comparison of technical pipelines: existing approaches vs. ours. (**a**) Traditional techniques often rely on a dual-branch framework that leverages independent backbones to separately encode features from images and depth inputs. (**b**) Lightweight approaches fuse image and depth features via separate convolutions, and then transform them into a unified representation for a shared CNN. (**c**) Our method merges the strengths of both approaches by concatenating image and depth data in batches, extracting features via a unified Transformer backbone, and enhancing them with a cross-modal channel-wise attention module.The blue pipeline corresponds to the image processing module, whereas the yellow pipeline corresponds to the depth module.

The objective of calibration is to estimate the transformation matrix that aligns the LiDAR and camera coordinate systems. Existing approaches are generally classified into two groups, offline calibration [7,8,9,10,11,12] and online calibration [13,14,15,16,17,18], as illustrated in Figure 1. Offline strategies typically rely on dedicated calibration targets and manual feature selection, which is labor-intensive and unable to automatically compensate for perturbations during operation. By contrast, online methods often substitute the handcrafted feature extraction step with learning-based techniques that identify salient structures directly from multi-modal sensor data. Consequently, online methods are proposed to conduct calibration in an automated manner. With the rapid progress of deep learning techniques, such as CNNs, numerous studies [13,14,18,19,20,21] have proposed supervised data-driven approaches to handle LiDAR–camera calibration task. Specifically, recent approaches treat calibration as a deep regression problem, aiming to estimate the 6-DoF (Degree of Freedom) extrinsic parameters between LiDAR and the camera using learning-based methods. Most of these methods adopt the general network structure illustrated in Figure 1a. The input typically consists of the original image paired with a misaligned depth map, and the pipeline is organized into two separate streams—one for image features and another for depth features—followed by feature extraction, correspondence computation, and parameter regression. Some of them also take the architecture shown in Figure 1b to design the network model, which projects the raw point cloud into related images for input generation; then they are extracted by a uniform CNN backbone to obtain uniform feature maps. Due to CNN’s strong fitting capacity, their performance has been facilitated significantly.The majority of methods perform calibration in a single-shot manner [22,23,24], whereas others employ multiple networks [13,14,19,20,21] for iterative refinement.

**Figure 2:**
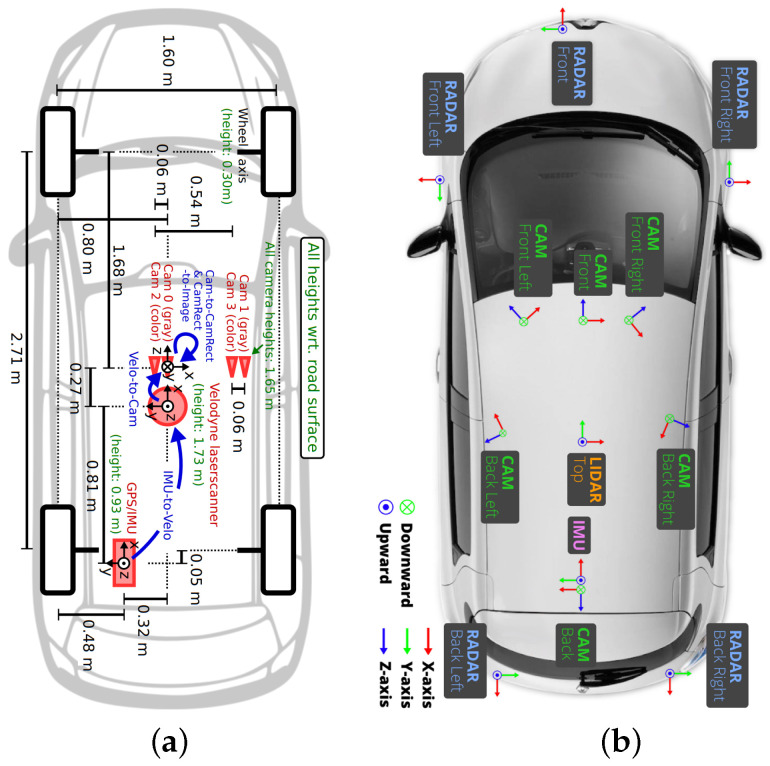
Comparison of multiple sensor configurations in mainstream autonomous driving datasets. (**a**) Sensor setups in the KITTI Dataset: single LiDAR sensor and four cameras. (**b**) Sensor configuration in the nuScenes dataset, featuring a single LiDAR sensor and six surround-view cameras.

Figure 2 illustrates the sensor configurations of two commonly used open-source datasets, KITTI [25] and nuScenes [26].

Although state-of-the-art deep learning-based methods have achieved remarkable progress, we argue that they still suffer from two fundamental limitations: (1) **One-to-one calibration**: In practical deployment scenarios—such as those illustrated in Figure 2a,b—autonomous platforms typically employ multi-camera and multi-LiDAR configurations. However, most existing calibration methods are designed for single LiDAR–camera pairs. Extending these approaches to multi-view settings (e.g., front, rear, left, and right) is non-trivial due to significant viewpoint variations, leading to poor generalization, increased calibration errors, and higher memory consumption. (2) **Inefficiencies in the calibration architecture**: Most traditional methods separate calibration into dual-stream networks, enabling modality-specific feature extraction but incurring high computational and memory costs, especially during iterative inference. In contrast, recent lightweight approaches unify inputs by projecting 3D point clouds as 2D depth channels, reducing overhead but struggling to fully exploit transformer-based backbones. Since transformers require large-scale data and are hard to train on limited samples, these methods often yield poor convergence and degraded calibration performance.

To overcome these two challenges, we introduce the following three-fold enhancement strategy:

- First, we adopt a large-scale pre-trained Swin Transformer as a unified backbone for joint feature extraction from both RGB (Red Green Blue) images and depth maps. Leveraging pre-trained weights significantly reduces the training cost and improves memory efficiency during iterative inference.
- Second, we design a cross-modal channel-wise attention mechanism that facilitates multi-scale feature integration between camera and LiDAR modalities. This mutually guided attention module enhances salient and correspondingly aligned features via context-aware reinforcement.
- Finally, we implement a configurable calibration head architecture that accommodates varying numbers of cameras. Each head independently predicts the extrinsics for its corresponding monocular image–depth map pair. This modular design avoids cross-view feature entanglement, effectively mitigating the artifacts caused by shared-head architectures and preserving viewpoint-specific calibration fidelity.

**Figure 3:**
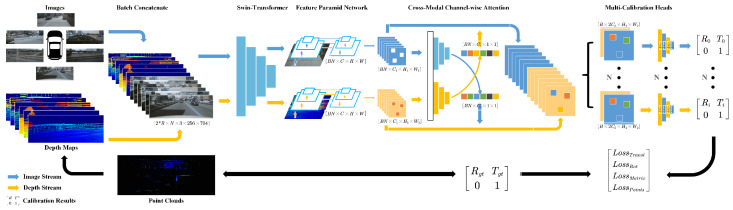
Our method estimates LiDAR–camera extrinsics as follows. Multi-view images from calibrated cameras and projected depth maps from miscalibrated LiDAR are concatenated and processed by a unified Swin Transformer backbone for feature extraction (The asterisk (*) denotes element-wise multiplication with the same dimension.). Multi-scale features are obtained through two trainable FPNs (Feature Pyramid NetworkS) and fused with a cross-modal channel-wise attention module. The fused features are passed to calibration heads that regress 6-DoF transformations $T_{pred}$, which represent the deviation between the initial extrinsics $T_{init}$ and the ground truth $T_{gt}$.

Figure 3 provides an overview of the full calibration workflow.

Results obtained on the nuScenes dataset [26] indicate that the proposed method performs slightly worse than state-of-the-art approaches in calibration accuracy while achieving significant model size reduction and supporting flexible calibration configurations beyond the conventional one-to-one setting. The main contributions of this work are summarized as follows:

- To address multi-sensor calibration, we present Multi-Calib, an online deep learning model with explicit depth supervision capable of aligning multiple LiDAR sensors and cameras.
- We introduce a cross-modal channel-wise attention module for mutually guided fusion between camera and LiDAR features, achieving highly efficient integration.
- A novel loss function is developed based on the intrinsic correlation between translation and rotation errors, effectively improving LiDAR–camera registration accuracy.

The structure and content of this article are summarized as follows: Section 2 primarily elaborates on relevant research works in the field. Section 3 systematically presents the network architecture and its underlying principles. Section 4 comprehensively documents experimental results, implementation details, and ablation studies. Section 5 summarizes the main findings and outlines directions for future research.

## 2. Related Work

There will be an introduction to the related works in terms of offline calibration and online calibration methods in this section.

### 2.1. Offline Calibration

Traditional offline approaches [8,9,10,27,28,29,30] obtain 2D–3D correspondences between point clouds and images by using manually arranged calibration targets, including chessboards or fiducial markers. Based on these geometric cues, the 6-DoF extrinsic parameters are solved via nonlinear least-squares optimization. Despite their accuracy, these approaches are limited by high operational costs, sensitivity to initial conditions, and strong reliance on human involvement. Additionally, such methods cannot adapt to dynamic conditions: over time, sensor misalignment may occur due to vibration or environmental factors, requiring frequent recalibration. These limitations severely hinder scalability and long-term deployment. To address this, recent efforts have shifted toward online, self-supervised calibration methods that avoid manual setup and enable real-time adaptation to pose drift.

### 2.2. Online Calibration

There are two types of existing online calibration methods: handcrafted and learning-based. Both of them will be introduced sequentially.

Handcrafted calibration methods are typically categorized into three main types: feature-based [23,31], motion-based [32,33,34], and mutual information-based approaches [35,36,37]. Feature-based methods rely on geometric correspondences—such as keypoints, edges, or planar surfaces—extracted from both LiDAR and camera data to estimate extrinsic parameters. Motion-based methods exploit temporal or multi-view consistency to recover the 6-DoF transformation between sensors. In contrast, mutual information-based methods optimize for statistical similarity, typically by aligning LiDAR intensity distributions with grayscale image patterns. While these traditional methods have shown effectiveness under controlled conditions, they share a common limitation: they are not end-to-end trainable and cannot jointly optimize all stages of the calibration pipeline. This fragmentation often leads to suboptimal performance and poor adaptability. To overcome these challenges, recent research has turned to learning-based solutions, which offer data-driven, end-to-end calibration frameworks with improved robustness and scalability.

The powerful representational capability of CNNs has been extensively exploited in learning-based calibration methods [13,14,19,20]. To accommodate CNN processing, LiDAR point clouds are projected onto 2D depth maps, and separate modality-specific CNNs extract features from the depth maps and camera RGB images. Subsequently, a number of MLPs (Multilayer Perceptrons) are employed to predict the 6-DoF extrinsic parameters of the LiDAR–camera setup. Other approaches [18,38] tackle calibration in a two-stage manner: geometric constraints, such as 2D–3D correspondences, are first identified via the CNN, followed by computation of the 6-DoF extrinsics. While one-shot methods [22] often struggle to achieve high accuracy, iterative strategies [21] and multi-network refinement approaches [14,19,20] have been proposed to progressively enhance calibration results across varying degrees of misalignment.

However, with the decreasing cost of sensors and the rapid advancement of autonomous driving technologies, most autonomous platforms are now equipped with multiple cameras and LiDAR sensors. Existing methods, however, are primarily designed for one-to-one calibration and are not well-suited for estimating extrinsics in many-to-many (multi-sensor) configurations. To overcome these limitations, we propose a cross-modal channel-wise attention module to deeply fuse and enhance multi-level features, along with configurable calibration heads capable of predicting multiple extrinsics between multiple cameras and LiDAR sensors within a single forward pass. Furthermore, a novel loss function is introduced to effectively guide the training of the calibration network.

## 3. Method

This work focuses on a mobile robotic platform equipped with multiple cameras and LiDAR sensors under the following assumptions: (i) Each LiDAR–camera pair has an overlapping field of view (FOV). (ii) Coarse initial extrinsics $T_{init}$ and camera intrinsics *K* are available.

### 3.1. Problem Formulation

Using multi-sensor inputs, the objective of the online calibration task is to refine the coarse initial estimate $T_{init_{i}}$ and recover the accurate extrinsics $T_{gt_{i}}$. Following previous works [14,19,20], to generate sufficient training data, a random transform matrix, $T_{rand}$, is applied to $T_{init_{i}}$ to obtain the ground truth $T_{gt_{i}}$. The miscalibrated depth map of the point cloud is then generated as

(1) $$Z_{c_{i}}\left(\begin{array}{c} u_{i}\\ v_{i}\\ 1 \end{array}\right)=K_{i}\left(T_{init_{i}}P\right)=K_{i}\left(R_{init_{i}}P+t_{init_{i}}\right)$$

(2) $$T_{gt_{i}}=T_{init_{i}}\times T_{rand}=\left(\begin{array}{cc} R_{gt_{i}} & t_{gt_{i}}\\ 0 & 1 \end{array}\right)$$

Here, $i\in \left[0,5\right]$ indexes the LiDAR–camera pairs, P=(X,Y,Z,1) represents a 3D LiDAR point, and $x=(u_{i},v_{i})$ denotes its corresponding 2D projection. $K_{i}$ stands for the camera intrinsic matrix, and $Z_{c_{i}}$ is an arbitrary depth-related scale factor. The initial transformation matrix $T_{init_{i}}$ comprises a rotation component, $R_{init_{i}}$, and a translation vector, $t_{init_{i}}$. The network receives a randomly miscalibrated depth map together with a resized RGB image as input, and the goal of online calibration is to estimate $T_{rand}$ to correct the extrinsic misalignment.

### 3.2. Network Architecture

**Figure 4:**
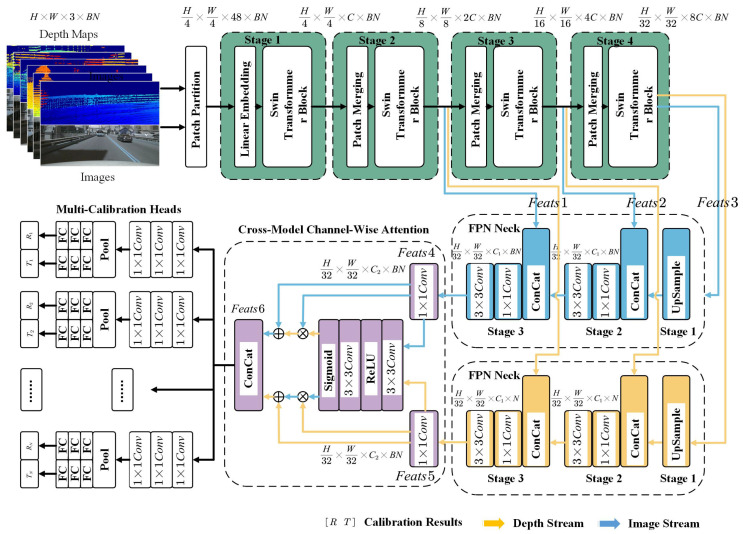
Multiple LiDAR–camera pairs are batched and fed into a frozen pretrained Swin Transformer backbone (shown in green). The resulting multi-level features (Feats1, Feats2, and Feats3) are forwarded to two trainable FPN necks—one for image features (blue) and one for depth features (yellow). The output features (Feats4 and Feats5) are then refined using a cross-modal channel-wise Attention block(shown in purple) to generate importance-weighted features, which are subsequently concatenated into Feats6. Finally, Feats6 is passed to the calibration heads to produce the predicted translation and rotation parameters.

The technical workflow of the proposed Multi-Calib network for LiDAR–camera calibration is illustrated in Figure 3, and a more detailed network subfigure is provided in Figure 4.

The system takes multiple images from different onboard cameras (top) and the corresponding depth maps projected from LiDAR point clouds (bottom) as input. To enhance robustness, the images are randomly scaled, while the point clouds undergo random affine transformations and shuffling. The resulting depth maps are further refined using a local window filter. These inputs are batch-concatenated and fed into a unified Transformer backbone for feature extraction using both modalities—unlike traditional dual-stream architectures that treat each modality separately. The extracted features are then processed by FPNs to obtain multi-scale representations, which are subsequently fused via a cross-modal channel-wise attention module. Finally, a configurable calibration head processes the fused features to output the 6-DoF extrinsic parameters for each LiDAR–camera pair in one forward pass.

### 3.3. Loss Functions

For a set of *N* RGB images and their corresponding initial depth maps, two loss terms are applied during training: a regression loss, $L_{T}$, and a point cloud distance loss, $L_{P}$, as formulated in [20].

(3) $$L=\left(1-\lambda_{P}\right)L_{T}+\lambda_{P}L_{P}$$

where $\lambda_{P}$ denotes the point cloud distance loss weight.

#### Regression Loss

For the predicted translation vector $t_{\mathrm{pred}}$, we apply the Huber loss function. As for the rotation loss $L_{q}$, we adopt the angular distance to quantify the discrepancy between them, as defined below:

(4) $$L_{R}=Distance_{a}\left(q_{gt},q_{pred}\right)$$

where $q_{gt}$ denotes the ground-truth quaternion, $q_{pred}$ is the predicted quaternion, and $Distance_{a}$ represents the angular distance between them.

However, it is important to emphasize that rotation and translation are not independent components; they are intrinsically coupled within the rigid-body transformation matrix. Ignoring this interdependence and optimizing their losses separately may lead to suboptimal calibration performance. To better capture this coupling and enhance the overall calibration accuracy, we introduce a matrix-based loss function, $L_{M}$, which directly penalizes the discrepancy between the predicted and ground-truth transform matrices, as defined below:

(5) $$L_{M}=\Sigma_{i=1}^{5}HuberLoss(T_{pred_{i}}\times inv\left(T_{rand_{i}}\right),E)$$

where $T_{pred_{i}}$ denotes the predicted transform matrix, which should ideally be the identity matrix *E* when multiplied by the inverse of the random transformation. The total regression loss $L_{T}$ is defined as the combination of the translation, rotation, and matrix losses:

(6) $$L_{T}=\lambda_{t}L_{t}+\lambda_{q}L_{R}+\lambda_{m}L_{M}$$

where $\lambda_{t}$, $\lambda_{q}$, and $\lambda_{m}$ denote the respective weights for the translation, rotation, and matrix loss terms.

## 4. Experiments

The proposed calibration approach is evaluated on the nuScenes dataset. This section describes dataset preparation and implementation details and presents an analysis of the experimental results.

### 4.1. Dataset Preparation

Dataset Generation: We validate our proposed network using the full nuScenes dataset [26], which contains 1000 multi-modal driving sequences captured with six cameras. We use the officially designated splits of the dataset: 700 sequences for training, 150 for validation, and 150 for testing. Each scene provides six RGB images from the front, front-left, front-right, rear, rear-left, and rear-right cameras, together with LiDAR point clouds obtained from the roof-mounted LiDAR sensor.

**Figure 5:**
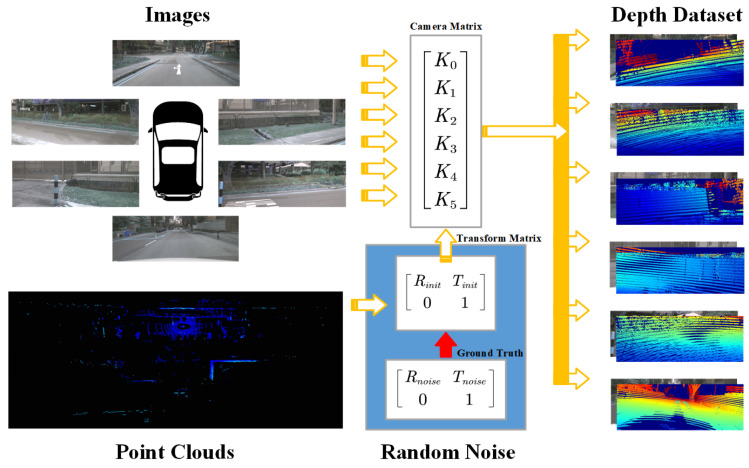
Dataset preparation process.

To simulate real-world calibration errors, we follow the protocol in [20] and apply a random affine transformation to the LiDAR point clouds. The transformation parameters are sampled from uniform distributions over translation ranges [−x,x] with x∈ 1.5 m, 1.0 m, 0.5 m, 0.25 m, 0.12 m and rotation ranges [−y,y] with $y\in 20^{\circ },\;15^{\circ },10^{\circ },\;5^{\circ },\;2^{\circ }$. The whole process is depicted in Figure 5. The range is selected based on our test results.

Evaluation Metrics: The experimental results are assessed in terms of both rotation and translation of the calibration parameters. The translation vector is quantified using the Euclidean distance, and its absolute error is computed as follows:

(7) $$E_{t}=\left|\right|t_{pred}-t_{gt}{\left|\right|}_{2}$$

Here, $\left|\right|\cdot {\left|\right|}_{2}$ represents the L2-norm of a vector. In addition, the absolute errors of the translation vector are evaluated separately along the X, Y, and Z axes.

### 4.2. Implementation Details

Training Details: The original image resolution is 1600×900, which is resized to 704×256 as the input to our model. The LiDAR sensor used in the nuScenes dataset is the Velodyne HDL-32E sensor (Velodyne LiDAR, San Jose, CA, USA), which features 32 laser channels and a $360^{\circ }$ horizontal field of view. Each LiDAR frame contains up to 55,000 points. Depth maps are generated using the camera intrinsics and the original LiDAR-to-camera extrinsics, and they are subsequently resized to 704×256.

We train the proposed network for 60 epochs using four NVIDIA RTX 4090 GPUs (NVIDIA Corporation, Santa Clara, CA, USA) with a batch size of 10. The AdamW optimizer is employed with an initial learning rate of $1\times 10^{-3}$. During the first 300 iterations, the learning rate is linearly warmed up using a small multiplicative factor. Then, it is halved every 10 epochs for the subsequent 50 epochs. Finally, a cosine annealing schedule is applied during the last 10 epochs.

To ensure sufficient point cloud density, we limit the point cloud range from [−54.0,−54.0,−5.0] to [54.0,54.0,5.0]. To maintain balanced optimization across the sub-tasks and ensure equal contribution from each objective during training, all loss function weights are set to 1.0, except for the point cloud distance loss, which is weighted at 0.5 to achieve equilibrium between the regression and point cloud distance components. For a fair comparison with LCCNet, the iterative step size is kept at five.

### 4.3. Comparison to State-of-the-Art Models

Baselines: Two different methods were selected as baselines, and their experimental settings were maintained for a fair comparison.

Comparison of basic parameters: Table 1 presents the model sizes of CMRNet [39], LCCNet [20], SCNet [40], and our proposed Multi-Calib model. To the best of our knowledge, no existing published and open-source methods are capable of calibrating multiple cameras simultaneously within a unified framework. Therefore, we compare our approach with representative single-camera calibration models, which remain the strongest competitors in the current literature. It can be seen that

- CMRNet uses the fewest iterations, but its three separate models do not share parameters, resulting in a total model size of 106.20 M—which is significantly larger than ours (78.79 M). In addition, our model is scalable to any number of LiDAR–camera pairs, which exceeds the capacity of all of them.
- Unlike LCCNet, which requires training five separate whole models for iterative refinement, our method adopts a unified frozen Swin Transformer as a shared backbone across all iterations, significantly reducing model size and the training cost. As shown in Table 1, the total size of our model is only 78.79 M, with just 5.99 M parameters requiring training. In contrast, LCCNet trains five independent models (66.75 M each), resulting in a total size exceeding 333.75 M.

Results: Table 2 presents the comparison with the baseline methods. Multi-Calib achieves performance comparable to all baselines across the tested environments, indicating that the combination of frozen shared backbones and the proposed iterative training strategy is effective, without relying on additional sensor data or multi-frame inputs.

**Figure 6:**
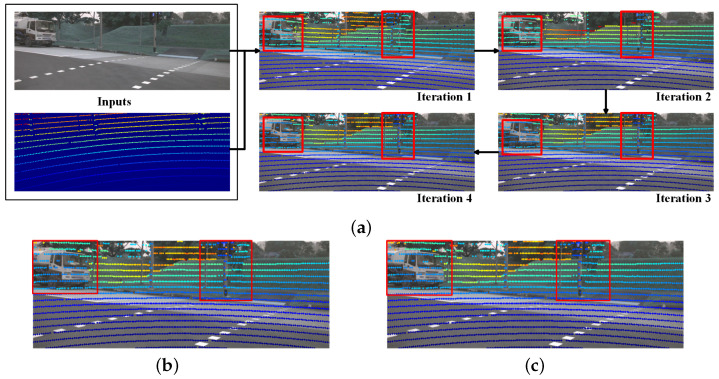
Visualization of the progressive refinement process from the initial input to the final ground truth. (**a**) Iteration Results: Each subfigure shows intermediate results. Arrows indicate forward refinement stages. (**b**) Visualization of predicted extrinsics after 4 iterations of network inference. (**c**) Ground Truth: The red boxes highlight the most noticeable differences.

Figure 6 illustrates the predicted depth maps and the results of the iterative calibration, providing an intuitive demonstration of Multi-Calib’s effectiveness.

### 4.4. Ablation Studies

#### 4.4.1. Backbone Selection

To validate our backbone network strategy, we first selected two representative multi-sensor fusion perception tasks: 3D object detection and BEV (Bird’s Eye View) map segmentation. For both tasks, we adopted BEVFusion [2], a multi-task perception model that integrates surround-view cameras and LiDAR. To simulate extrinsic errors caused by long-term platform operation and vibrations, we injected average noise into the extrinsic matrices, following the design methodology of prior work. The corresponding perception results are summarized in Table 3, where the NDS (nuScenes Detection Score) and mAP (mean Average Precision) are reported for 3D object detection, and the mIoU (Mean Intersection over Union) is used as the evaluation metric for BEV map segmentation.

In the setting without noise injection (w/o Noise), which corresponds to using ground-truth extrinsics, BEVFusion achieves an NDS of 71.15 and an mAP of 68.41 on the 3D object detection task. For BEV map segmentation, the best performance is observed in the Drivable Area, with an mIoU as high as 83.04.

In the first experiment, we injected average noise values of ±0.12 m in translation and ±1° in rotation. Under this setting, both the NDS and mAP exhibited a slight decrease of 2.08 and 2.68, respectively. For the BEV map segmentation task, degradation was relatively minor, with the most significant drop observed in the Carpark Area (↓ 7.05).

In the second experiment, when the noise level was increased to ±0.5 m/$\pm 10^{\circ }$, BEVFusion suffered a sharp performance decline. On the 3D object detection task, the NDS dropped to 49.69 (one-third lower), while the mAP decreased by nearly half (↓ 31.40). The BEV map segmentation task also experienced substantial degradation, with the Carpark Area decreasing by 26.63 and Divider by 22.91, resulting in an average drop of 17.03.

In the third experiment, with noise increased to ±1.5 m/$\pm 20^{\circ }$, the 3D object detection task almost completely lost detection capability, with the mAP reduced to 5.64 and the NDS to 26.44. Meanwhile, BEV map segmentation showed an average drop of 32.85, where the Carpark Area decreased the most (↓ 60.13), while the Drivable Area decreased the least (↓ 19.53).

These results clearly demonstrate that extrinsic errors have a critical impact on multi-sensor fusion perception. As the error magnitude increases, perception accuracy consistently degrades, exhibiting a gradual decline at lower noise levels and a drastic collapse at higher noise levels. Therefore, we argue that the primary goal of online calibration is to constrain extrinsic errors within ±0.12 m/$\pm 1^{\circ }$ while maintaining model efficiency.

To further investigate the impact of backbone networks on LiDAR–camera calibration, we designed a second experiment comparing representative backbone architectures commonly used in calibration models. The evaluation was divided into two groups: (1) fully frozen backbones, where all backbone parameters are fixed (this setting is advantageous for multi-round iterative calibration since it significantly reduces memory consumption and allows for larger batch sizes, thereby improving training efficiency), and (2) non-frozen backbones, including fully trainable networks and a partially frozen Swin Transformer (with only the final output layer unfrozen).

The experimental setup was as follows: translation noise: ±1.5 m, rotation noise: $\pm 20^{\circ }$, and training conducted on four GTX 4090 GPUs(NVIDIA Corporation, Santa Clara, CA, USA). According to the available GPU memory, the batch size was set to 24 for frozen backbones, while for non-frozen backbones, it was 4 for ResNet50, 3 for ResNet101, and 2 for the Swin Transformer. Training was performed for 60 epochs, with an initial learning rate of 0.001, which was halved every 5 epochs. The final calibration results are reported in Table 4.

From the first-round calibration results, the partially frozen Swin Transformer achieves the best performance, with an average translation error of 19.41 cm and an average rotation error of $2.18^{\circ }$. The second-best results are obtained by the trainable ResNet50 model and the trainable Swin Transformer, while the fully frozen ResNet101 and ResNet50 models yield the worst performance.

From the perspective of iterative calibration, the frozen Swin Transformer demonstrates the highest efficiency, requiring only 78.79 M parameters over five iterations, thereby minimizing memory usage and training time. Although the partially frozen Swin Transformer achieves the best accuracy, it requires 130 M parameters, which represents a 64% increase compared to the fully frozen variant. The trainable ResNet50 model ranks second in terms of accuracy but demands 148 M parameters, an 88% increase, while providing only marginal improvements of 1.01 cm in translation error and $0.08^{\circ }$ in rotation error.

We attribute these results to the inherent advantages of the Swin Transformer’s global attention mechanism for calibration tasks. Since most of its parameters are concentrated in the latter layers, fully training the backbone is suboptimal given the limited size of the nuScenes dataset. In contrast, partial freezing balances stability and adaptability, thereby achieving the best accuracy. However, considering the significant growth in parameter size with only limited accuracy gains, we conclude that adopting a fully frozen Swin Transformer backbone represents the most balanced and efficient choice.

#### 4.4.2. Module Ablation

To evaluate the effectiveness of each proposed module, we conduct an ablation study at the first calibration iteration under a challenging miscalibration range of [−1.5m,1.5m]/$\left[-20^{\circ },20^{\circ }\right]$, as shown in Table 5. Starting from a baseline model without any enhancement modules (FPN, CCFusion, or Matrix Loss), we progressively introduce each component to assess its individual and joint contributions.

Adding the FPN module alone slightly improves rotation accuracy (e.g., $Rot_{X}$ decreases by $0.23^{\circ }$) and reduces translation errors along the *Y*-axis by 5.22cm, though some metrics (e.g., $T_{X}$) increase slightly, indicating limited benefits when used in isolation.

Introducing the CCFusion module significantly enhances performance across all metrics. Translation errors along all three axes are notably reduced (e.g., $T_{X}$ by 9.59cm, $T_{Z}$ by 7.89cm, and $T_{Z}$ by 7.11cm), and rotation accuracy improves as well (e.g., $Rot_{Z}$ decreases by $1.31^{\circ }$). These improvements validate the effectiveness of the cross-modal fusion model in aligning image and depth features.

Further incorporating the Matrix Loss leads to the best overall performance across both translation and rotation metrics. Compared to the baseline, the translation error along the *X*-axis is reduced from 38.98cm to 24.06cm, and the rotation error on the *X*-axis drops significantly from $3.64^{\circ }$ to $1.43^{\circ }$, yielding a $2.21^{\circ }$ improvement. This substantial reduction highlights the effectiveness of enforcing geometric consistency between translation and rotation predictions. By regularizing the network through matrix-level constraints, the model achieves more stable and accurate extrinsic estimation, particularly under large initial miscalibrations.

In summary, all three modules exhibit strong complementary effects. Their combined application achieves the highest calibration accuracy and validates the design of our full model.

#### 4.4.3. Calibration Pairs Analysis

To highlight the capability of our Multi-Calib network in simultaneously calibrating multiple LiDAR–camera pairs, we conducted experiments on the nuScenes dataset using different numbers of sensor pairs. The injected perturbations were kept consistent with the previous settings, with miscalibration ranges of [−1.5 m, 1.5 m] and [−20°, 20°]. The detailed calibration performance under each configuration is summarized in the Table 6.

The experimental results show that the calibration accuracy remains highly consistent across different numbers of LiDAR–camera pairs. Both translation and rotation errors exhibit only small variations, and no performance degradation trend is observed as the number of calibrated pairs increases.

This behavior can be explained by the network design: the Multi-Calib framework allocates an independent calibration head to each LiDAR–camera pair, and all pairs share a common feature extraction backbone. As a result, increasing the number of pairs mainly adds parameters to the pair-specific calibration heads but does not alter the core feature representation or the learning dynamics of the backbone.

Consequently, the increase in calibration pairs has minimal influence on overall accuracy. The results indicate that the system maintains stable and reliable calibration performance even at the largest tested configuration, demonstrating strong scalability and robustness for multi-sensor calibration.

## 5. Conclusions

This paper proposes a lightweight, learning-based method for LiDAR–camera extrinsic calibration. Unlike existing approaches that are limited to fixed sensor configurations, our model supports flexible calibration of multiple LiDAR–camera pairs, making it adaptable to various autonomous driving platforms. We adopt a unified Transformer backbone to extract features from both RGB images and LiDAR depth maps, enabling efficient end-to-end learning with reduced computational costs. A cross-modal channel-wise attention module is introduced to enhance feature fusion between modalities. To handle varying sensor setups, we design configurable calibration heads that adjust to the number of sensor pairs and support simultaneous prediction in a single forward pass. Additionally, a novel loss function jointly optimizes translation and rotation by modeling their correlation, improving calibration stability and accuracy. Extensive experiments on the nuScenes dataset validate the effectiveness and scalability of our approach.

## Figures and Tables

**Table 1:** Comparison of model size, iterations, and camera num.

Method	SCNet	CMRNet	LCCNet	Ours
Per model size (M)	-	**35.40**	66.75	54.83
Trainable Params	-	35.40	66.75	**5.99**
Total size	-	106.20	333.75	**78.79**
Iterations	5	**3**	5	5
Camera num	1	1	1	**6** ^1^

^1^ Although nuScenes provides only 6 cameras, our model is capable of handling additional cameras with a negligible increase in parameters. The values in bold denote the optimal parameters.

**Table 2:** Comparison of calibration methods under a miscalibration range of [−1.5 m, 1.5 m]/[−20°, 20°] in nuScenes.

Method	Transl Error (cm)			Rot Error (°)		
	Mean	Median	Std	Mean	Median	Std
LCCNet	2.006	1.643	1.415	0.215	0.169	0.187
SCNet	1.368	1.212	0.975	0.148	0.129	0.122
Ours	2.651	2.168	3.401	0.246	0.1840	0.347

**Table 3:** Performance comparison of 3D object detection and BEV map segmentation under different noise levels.

Tasks	Metrics	w/o Noise	±0.12 m/±1°	±0.5 m/±10°	±1.5 m/±20°
3D Object-Det [20]	NDS	71.15	69.07 (↓ 2.08)	49.69 (↓ 21.46)	26.44 (↓ 44.71)
	mAP	68.41	65.73 (↓ 2.68)	37.01 (↓ 31.40)	5.64 (↓ 62.77)
BEV Map-Seg [20]	mIoU	w/o Noise	±0.12m/±1°	±0.5m/±10°	±1.5m/±20°
	Drivable Area	83.04	82.35 (↓ 0.69)	74.66 (↓ 8.38)	63.51 (↓ 19.53)
	Ped Crossing	58.56	57.64 (↓ 0.92)	44.20 (↓ 14.36)	18.73 (↓ 39.83)
	Walkway	57.81	55.86 (↓ 1.95)	39.01 (↓ 18.80)	19.59 (↓ 38.22)
	Stop Line	47.61	46.75 (↓ 0.86)	36.52 (↓ 11.09)	12.56 (↓ 35.05)
	Carpark Area	77.58	70.53 (↓ 7.05)	50.95 (↓ 26.63)	17.45 (↓ 60.13)
	Divider	48.42	44.77 (↓ 3.65)	25.51 (↓ 22.91)	14.73 (↓ 33.69) ^2^

^2^ The downward arrow indicates the performance change compared to the without-noise situation, and the red text is used for emphasis.

**Table 4:** Comparison of backbone freezing strategies under a miscalibration range of [−1.5 m, 1.5 m]/[−20°, 20°] in nuScenes.

Backbone	Transl Error (cm)			Rot Error (°)		
	X	Y	Z	Rot	Pitch	Yaw
ResNet50 (Frozen) [41]	41.09	53.47	19.11	1.81	2.07	5.31
ResNet101 (Frozen) [41]	41.67	51.41	19.32	1.81	2.07	5.42
Swin-Transformer (Frozen) [42]	**24.06**	**26.90**	**20.43**	**1.43**	**1.64**	**4.37**
ResNet50 (Trainable)	23.52	27.49	17.37	1.50	1.76	3.94
ResNet101 (Trainable)	24.91	29.11	19.04	1.54	1.84	4.11
Swin-Transformer (Trainable)	26.52	32.17	24.75	1.98	2.34	4.75
Swin-Transformer (Partially)	**20.36**	**23.90**	**13.97**	**1.29**	**1.48**	**3.78** ^3^

^3^ The bold text indicates the best performance.

**Table 5:** Ablation study on module effectiveness at the first iteration under a miscalibration range of [−1.5 m, 1.5 m]/[−20°, 20°] in nuScenes.

FPN	CCFusion	Matrix Loss	${\mathrm{Transl}}_{X}$ ${\mathrm{Transl}}_{Y}$ (cm) ${\mathrm{Transl}}_{Z}$	${\mathrm{Rot}}_{X}$ ${\mathrm{Rot}}_{Y}$ (°) ${\mathrm{Rot}}_{Z}$
no	no	no	38.98 45.47 27.82	3.64 3.80 7.00
yes	no	no	39.42 **(+0.40)** 40.25 **(−5.22)** 27.54 **(−0.28)**	3.41 **(−0.23)** 3.76 **(−0.04)** 7.06 **(+0.06)**
yes	yes	no	29.83 **(−9.59)** 32.36 **(−7.89)** 20.43 **(−7.11)**	3.03 **(−0.38)** 3.19 **(−0.57)** 5.69 **(−1.37)**
yes	yes	yes	24.06 **(−5.80)** 26.90 **(−5.46)** 16.64 **(−3.79)**	1.43 **(−1.60)** 1.64 **(−1.55)** 4.37 **(−1.32)** ^4^

^4^ Red denotes an increase in error, whereas green denotes a decrease in error.

**Table 6:** Translation and rotation errors across six calibration pairs at the first iteration under a miscalibration range of [−1.5 m, 1.5 m]/[−20°, 20°] in nuScenes.

Errors/Pairs	Axis	1	2	3	4	5	6
Translation (cm)	X	26.05	25.75	24.99	26.62	26.52	**20.36**
	Y	**15.65**	22.76	25.01	24.72	27.28	23.90
	Z	**12.89**	15.81	16.27	16.51	17.50	13.97
Rotation (°)	X	1.35	1.56	1.62	1.62	1.68	**1.29**
	Y	**1.43**	1.73	1.77	1.79	1.88	1.47
	Z	**3.66** ^5^	4.51	4.63	4.57	4.78	3.78

^5^ The bold text indicates the best performance.

## Data Availability

The data used to support the finding of this study are available from the corresponding author upon request.

## References

[1] Song Z., Yang L., Xu S., Liu L., Xu D., Jia C., Jia F., Wang L. (2024). GraphBEV: Towards robust BEV feature alignment for multi-modal 3D object detection. Proceedings of the European Conference on Computer Vision (ECCV).

[2] Liu Z., Tang H., Amini A., Yang X., Mao H., Rus D.L., Han S. BEVFusion: Multi-task multi-sensor fusion with unified bird’s-eye view representation. Proceedings of the IEEE International Conference on Robotics and Automation (ICRA).

[3] Li Y., Yu A.W., Meng T., Caine B., Ngiam J., Peng D., Shen J., Lu Y., Zhou D., Le Q.V. Deepfusion: Lidar-camera deep fusion for multi-modal 3D object detection. Proceedings of the IEEE/CVF Conference on Computer Vision and Pattern Recognition.

[4] Zhao L., Zhou H., Zhu X., Song X., Li H., Tao W. (2023). LIF-Seg: LiDAR and camera image fusion for 3D LiDAR semantic segmentation. IEEE Trans. Multimed..

[5] Gu S., Yang J., Kong H. A cascaded LiDAR-camera fusion network for road detection. Proceedings of the IEEE International Conference on Robotics and Automation (ICRA).

[6] Fan R., Wang H., Cai P., Liu M. (2020). SNE-RoadSeg: Incorporating Surface Normal Information into Semantic Segmentation for Accurate Freespace Detection. Proceedings of the European Conference on Computer Vision (ECCV).

[7] Zhang J., Liu Y., Wen M., Yue Y., Zhang H., Wang D. L2V2T2-Calib: Automatic and unified extrinsic calibration toolbox for different 3D LiDAR, visual camera and thermal camera. Proceedings of the IEEE Intelligent Vehicles Symposium (IV).

[8] Yan G., He F., Shi C., Wei P., Cai X., Li Y. Joint camera intrinsic and LiDAR-camera extrinsic calibration. Proceedings of the IEEE International Conference on Robotics and Automation (ICRA).

[9] Jiao J., Chen F., Wei H., Wu J., Liu M. (2023). LCE-Calib: Automatic LiDAR-frame/event camera extrinsic calibration with a globally optimal solution. IEEE/ASME Trans. Mechatron..

[10] Lai Z., Wang Y., Guo S., Meng X., Li J., Li W., Han S. (2022). Laser reflectance feature assisted accurate extrinsic calibration for non-repetitive scanning LiDAR and camera systems. Opt. Express.

[11] Beltrán J., Guindel C., De La Escalera A., García F. (2022). Automatic extrinsic calibration method for LiDAR and camera sensor setups. IEEE Trans. Intell. Transp. Syst..

[12] Domhof J., Kooij J.F., Gavrila D.M. (2021). A joint extrinsic calibration tool for radar, camera and LiDAR. IEEE Trans. Intell. Veh..

[13] Xiao Y., Li Y., Meng C., Li X., Ji J., Zhang Y. CalibFormer: A transformer-based automatic LiDAR-camera calibration network. Proceedings of the IEEE International Conference on Robotics and Automation (ICRA).

[14] Zhu J., Xue J., Zhang P. CalibDepth: Unifying depth map representation for iterative LiDAR-camera online calibration. Proceedings of the IEEE International Conference on Robotics and Automation (ICRA).

[15] Sun Y., Li J., Wang Y., Xu X., Yang X., Sun Z. (2022). ATOP: An attention-to-optimization approach for automatic LiDAR-camera calibration via cross-modal object matching. IEEE Trans. Intell. Veh..

[16] Ren S., Zeng Y., Hou J., Chen X. (2022). CorrI2P: Deep image-to-point cloud registration via dense correspondence. IEEE Trans. Circuits Syst. Video Technol..

[17] Liao Y., Li J., Kang S., Li Q., Zhu G., Yuan S., Dong Z., Yang B. (2023). SE-Calib: Semantic edge-based LiDAR–camera boresight online calibration in urban scenes. IEEE Trans. Geosci. Remote Sens..

[18] Jeon Y., Seo S.W. (2022). EFGHNet: A versatile image-to-point cloud registration network for extreme outdoor environment. IEEE Robot. Autom. Lett..

[19] Yuan K., Guo Z., Wang Z.J. (2020). RGGNet: Tolerance aware LiDAR-camera online calibration with geometric deep learning and generative model. IEEE Robot. Autom. Lett..

[20] Lv X., Wang B., Dou Z., Ye D., Wang S. LCCNet: LiDAR and camera self-calibration using cost volume network. Proceedings of the IEEE/CVF Conference on Computer Vision and Pattern Recognition (CVPR).

[21] Zhu A., Xiao Y., Liu C., Tan M., Cao Z. (2024). Lightweight LiDAR-camera alignment with homogeneous local-global aware representation. IEEE Trans. Intell. Transp. Syst..

[22] Shi J., Zhu Z., Zhang J., Liu R., Wang Z., Chen S., Liu H. CalibRCNN: Calibrating camera and LiDAR by recurrent convolutional neural network and geometric constraints. Proceedings of the IEEE/RSJ International Conference on Intelligent Robots and Systems (IROS).

[23] Ye C., Pan H., Gao H. (2021). Keypoint-based LiDAR-camera online calibration with robust geometric network. IEEE Trans. Instrum. Meas..

[24] Lv X., Wang S., Ye D. (2021). CFNet: LiDAR-camera registration using calibration flow network. Sensors.

[25] Geiger A., Lenz P., Urtasun R. Are we ready for autonomous driving? The KITTI vision benchmark suite. Proceedings of the 2012 IEEE Conference on Computer Vision and Pattern Recognition (CVPR).

[26] Caesar H., Bankiti V., Lang A.H., Vora S., Liong V.E., Xu Q., Krishnan A., Pan Y., Baldan G., Beijbom O. nuScenes: A multimodal dataset for autonomous driving. Proceedings of the IEEE/CVF Conference on Computer Vision and Pattern Recognition (CVPR).

[27] Zhou L., Li Z., Kaess M. Automatic extrinsic calibration of a camera and a 3D lidar using line and plane correspondences. Proceedings of the 2018 IEEE/RSJ International Conference on Intelligent Robots and Systems (IROS).

[28] Pusztai Z., Hajder L. Accurate Calibration of LiDAR-Camera Systems Using Ordinary Boxes. Proceedings of the IEEE International Conference on Computer Vision Workshops (ICCVW).

[29] Tsai D., Worrall S., Shan M., Lohr A., Nebot E. Optimising the Selection of Samples for Robust LiDAR-Camera Calibration. Proceedings of the 2021 IEEE International Intelligent Transportation Systems Conference (ITSC).

[30] Kim E.s., Park S.Y. (2019). Extrinsic calibration between camera and LiDAR sensors by matching multiple 3D planes. Sensors.

[31] Yuan C., Liu X., Hong X., Zhang F. (2021). Pixel-level extrinsic self calibration of high resolution LiDAR and camera in targetless environments. IEEE Robot. Autom. Lett..

[32] Ou N., Cai H., Yang J., Wang J. (2023). Targetless extrinsic calibration of camera and low-resolution 3-D LiDAR. IEEE Sens. J..

[33] Tu D., Wang B., Cui H., Liu Y., Shen S. Multi-camera-LiDAR auto-calibration by joint structure-from-motion. Proceedings of the IEEE/RSJ International Conference on Intelligent Robots and Systems (IROS).

[34] Peršić J., Petrović L., Marković I., Petrović I. (2021). Spatiotemporal multisensor calibration via Gaussian processes moving target tracking. IEEE Trans. Robot..

[35] Pandey G., McBride J.R., Savarese S., Eustice R.M. (2015). Automatic extrinsic calibration of vision and lidar by maximizing mutual information. J. Field Robot..

[36] Pandey G., McBride J., Savarese S., Eustice R. Automatic targetless extrinsic calibration of a 3D lidar and camera by maximizing mutual information. Proceedings of the Twenty-Sixth AAAI Conference on Artificial Intelligence.

[37] Jiang P., Osteen P., Saripalli S. SemCal: Semantic LiDAR–Camera Calibration Using Neural Mutual Information Estimator. Proceedings of the 2021 IEEE International Conference on Multisensor Fusion and Integration for Intelligent Systems (MFI).

[38] Park C., Moghadam P., Kim S., Sridharan S., Fookes C. (2020). Spatiotemporal camera-LiDAR calibration: A targetless and structureless approach. IEEE Robot. Autom. Lett..

[39] Cattaneo D., Vaghi M., Ballardini A.L., Fontana S., Sorrenti D.G., Burgard W. CMRNet: Camera to LiDAR-map registration. Proceedings of the IEEE Intelligent Transportation Systems Conference (ITSC).

[40] Duan Z., Hu X., Ding J., An P., Huang X., Ma J. (2023). A robust LiDAR-camera self-calibration via rotation-based alignment and multi-level cost volume. IEEE Robot. Autom. Lett..

[41] He K., Zhang X., Ren S., Sun J. Deep Residual Learning for Image Recognition. Proceedings of the IEEE Conference on Computer Vision and Pattern Recognition (CVPR).

[42] Liu Z., Lin Y., Cao Y., Hu H., Wei Y., Zhang Z., Lin S., Guo B. Swin Transformer: Hierarchical Vision Transformer Using Shifted Windows. Proceedings of the IEEE/CVF International Conference on Computer Vision (ICCV).

